# Heterotopic Ossification in an Appendiceal Sessile Serrated Lesion With Synchronous Neuroendocrine Tumor

**DOI:** 10.14309/crj.0000000000001947

**Published:** 2025-12-24

**Authors:** Paul J. Wurtz, Douglas Walton, Calen Gist, Devin Moore, Serena Murphy, Carl Kay

**Affiliations:** 1 Department of Internal Medicine, Brooke Army Medical Center, San Antonio, TX; 2 Department of Pathology, Brooke Army Medical Center, San Antonio, TX; 3 Department of Surgery, Brooke Army Medical Center, San Antonio, TX; 4 Department of Gastroenterology, Carl R. Darnall Army Medical Center, Fort Hood, TX

**Keywords:** sessile serrated lesion, heterotopic ossification, appendiceal orifice, neuroendocrine tumor, osseous metaplasia, underwater cold snare, colorectal polyp

## Abstract

Heterotopic ossification (HO) in colorectal polyps is rare and scarcely reported in serrated lesions. We report an appendiceal sessile serrated lesion (SSL) with mature bone and a contiguous well-differentiated neuroendocrine tumor. A 64-year-old woman had a firm appendiceal-orifice polyp biopsied in 2012 showing calcification; SSL was identified in 2019. In 2024, an 8-mm lesion was removed en bloc by underwater cold snare; histology showed SSL with HO. Laparoscopic appendectomy confirmed residual SSL with HO and an adjacent 4.5-mm grade-1 neuroendocrine tumor (INSM1+, Ki-67 <3%) with negative margins. HO recognition should prompt en bloc excision and attentive pathology.

## INTRODUCTION

Heterotopic ossification (HO) is the formation of mature bone in ectopic soft tissue. It is uncommon in the gastrointestinal tract and is classically linked to mucin-rich neoplasia.^1^ Reports in serrated colorectal lesions are rare; to our knowledge, only 2 cases describe HO within a sessile serrated lesion (SSL), and only one involved the appendiceal orifice.^2^ Beyond SSLs, HO has been documented across other gastrointestinal neoplasms and settings, which provides context for osseous metaplasia in the colon and appendix.^3^ We present a recurrent, firm appendiceal-orifice SSL containing HO with a synchronous well-differentiated neuroendocrine tumor (NET). The case illustrates practical cues that should prompt complete en bloc excision and meticulous histopathology.

## CASE REPORT

A 64-year-old woman underwent routine colorectal cancer screening at age 53. She had no history of abdominal surgery, trauma, or genetic conditions predisposing to ectopic bone formation. In 2012, colonoscopy identified a firm, fungating 4-mm lesion at the appendiceal orifice. Cold-forceps sampling showed normal colonic mucosa with foci of dystrophic (osseous) calcification; complete polyp resection was not attempted. No endoscopic mucosal resection was attempted, and no injected lifting agent was used. In 2013, repeat colonoscopy with cold-forceps polypectomy again demonstrated persistent submucosal dystrophic calcification without dysplasia and without serrated features. In 2019, surveillance colonoscopy with cold-forceps sampling of a recurrent diminutive polyp at the same site revealed a SSL without evidence of HO.

**Figure 1.**
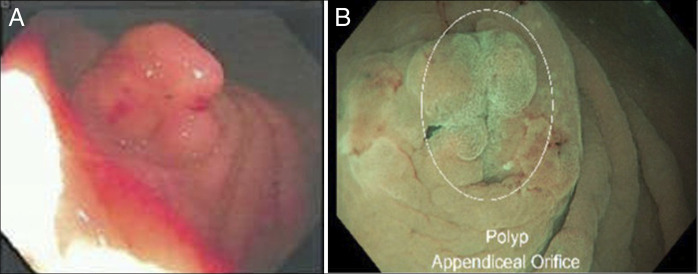
(A) Colonoscopy in 2012 showing a 4-mm firm lesion at the appendiceal orifice; forceps biopsy revealed dystrophic calcification. (B) Underwater cold-snare polypectomy in 2024 with en bloc removal of an 8-mm recurrent appendiceal-orifice lesion.

**Figure 2.**
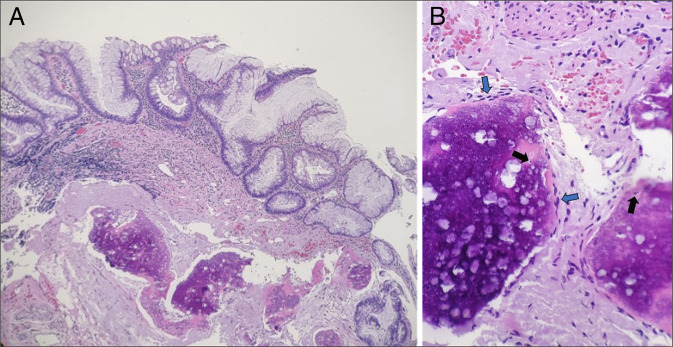
(A) Hematoxylin and eosin stain, 100×. Sessile serrated lesion overlying osseous metaplasia (heterotopic ossification) at the appendiceal orifice. (B) Hematoxylin and eosin stain, 400×. High-power view of osseous metaplasia showing osteocytes (black arrows) and osteoblast rimming (blue arrows).

In 2024, an 8-mm appendiceal-orifice lesion was removed en bloc using underwater cold-snare polypectomy without electrosurgery or a lifting agent (Figure 1); the earlier endoscopic appearance of the appendiceal-orifice lesion is shown for comparison (Figure 1). Histologic examination of the polypectomy specimen demonstrated serrated crypts with abundant mucin and focal expansion of crypt bases (Figure 2). Within the submucosa, and focally within the mucosa, there were mature bony trabeculae containing osteocytes in lacunae with adjacent osteoblast rimming and associated calcifications (Figure 2). A mild lymphocytic-predominant infiltrate with scattered eosinophils was present. These findings supported a diagnosis of SSL with HO.

**Figure 3.**
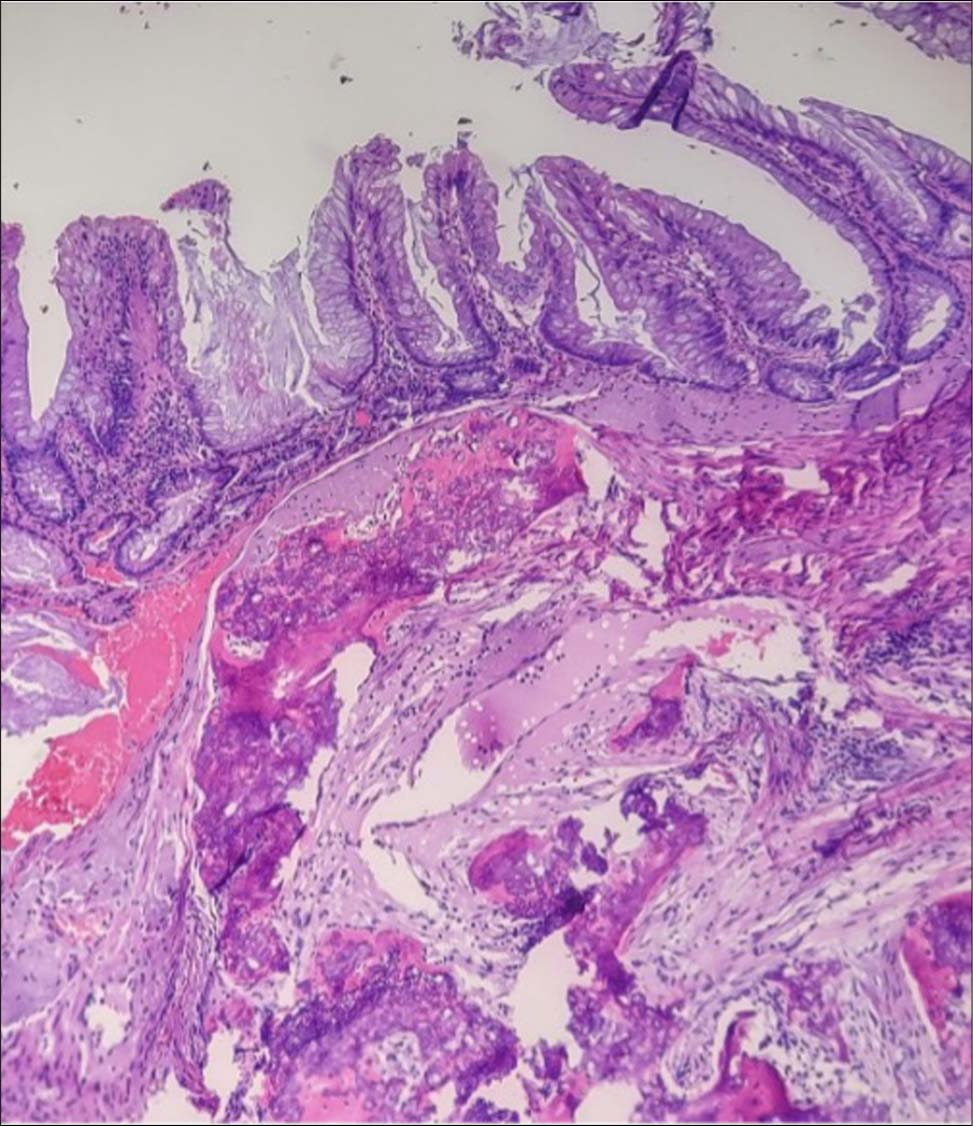
Hematoxylin and eosin stain, 100×. Appendectomy specimen confirming residual sessile serrated lesion with associated osseous metaplasia.

**Figure 4.**
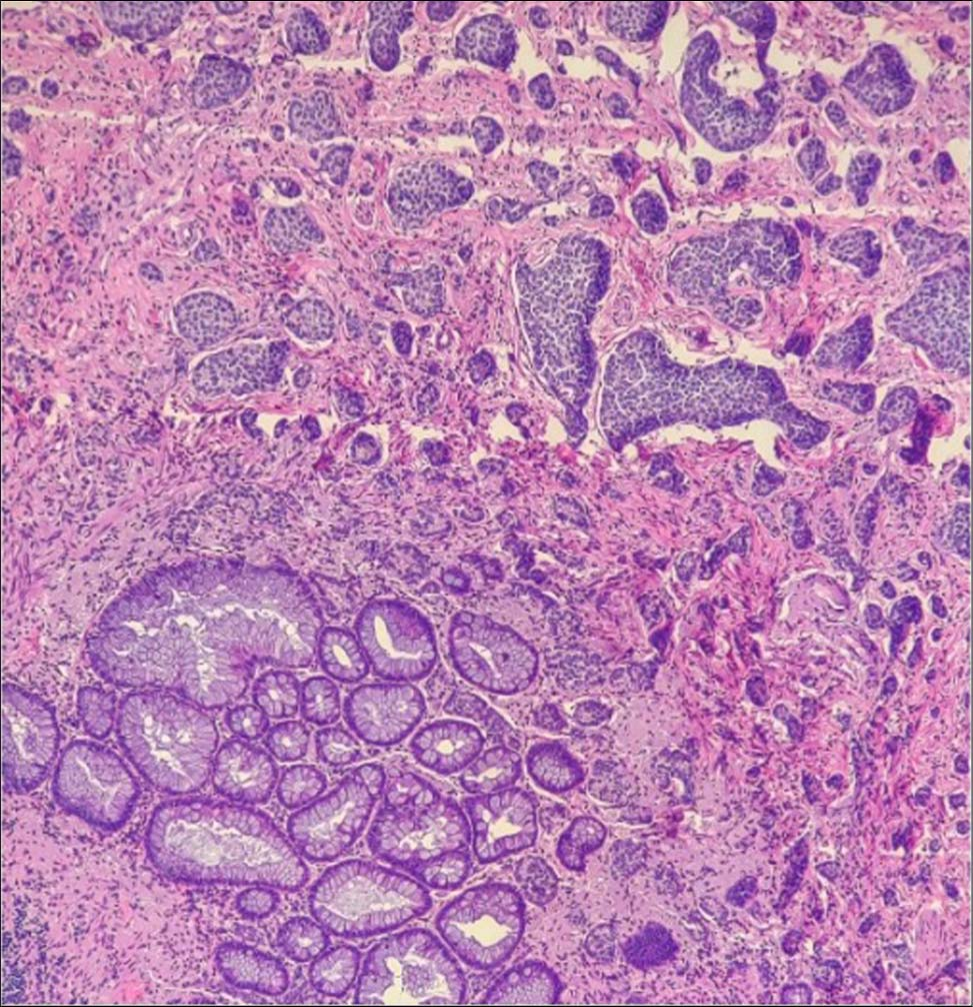
Hematoxylin and eosin stain, 100×. Well-differentiated neuroendocrine tumor (upper right) adjacent to residual sessile serrated lesion (lower left); INSM1 positive, Ki-67 <3%.

Approximately 2 months later, laparoscopic appendectomy with a cecal cuff was performed to definitively remove any residual lesion, given the protracted history of recurrence despite prior sampling, the firmness and submucosal ossification noted endoscopically and histologically, and the anatomically constrained location that increases the risk of incomplete endoscopic resection. Surgical pathology confirmed residual SSL with HO measuring 1.5 cm (Figure 3). Immediately adjacent to the SSL, invasive nests of monotonous cells with finely granular (“speckled”) chromatin were identified and were immunoreactive for INSM1 with a low Ki-67 index (<3%), diagnostic of a well-differentiated NET, WHO grade 1, measuring up to 0.45 cm (Figure 4). The proximal margin was negative for both the NET and the SSL. No dysplasia was identified elsewhere. The postoperative course was uneventful. At 6-month follow-up, the patient was asymptomatic, and a 1-year surveillance colonoscopy was planned.

## DISCUSSION

Fewer than 100 gastrointestinal cases of HO have been described, with a strong association to mucin-producing neoplasms.^1,3^ Within serrated pathology, HO is rare. Only 2 reports describe HO in SSLs, and only one was located at the appendiceal orifice.^2^ This case adds the first documented coexistence of HO within an SSL adjacent to a small well-differentiated NET, with no prior reports of HO occurring in association with a gastrointestinal NET identified in the literature, which broadens the spectrum of lesions that can develop ossification at this site. The 4.5-mm grade-1 NET appears incidental and pathogenetically unrelated to the ossification process. Its small size, low Ki-67, and negative margins carry an excellent prognosis and do not alter standard SSL surveillance.

Pathogenesis is incompletely understood. Extravasated mucin may promote production of bone morphogenetic proteins (BMPs) such as BMP-2, BMP-4, and BMP-9, which drive mesenchymal stromal cells toward osteoblast differentiation and osteoid deposition.^4^ The constrained appendiceal orifice may amplify mechanical and microenvironmental cues, and repeated partial resections may sustain osteogenic signaling. HO has been reported in mucinous adenocarcinomas and in traditional serrated adenomas, in appendiceal mucinous neoplasms, and in ulcerating tubular adenomas, which suggests that several colorectal histologies can undergo osseous metaplasia.^5–7^

Although the 2024 polypectomy specimen was removed en bloc, the decision to proceed with laparoscopic appendectomy was influenced by (i) the 12-year history of recurrent/residual lesion despite multiple biopsies, (ii) the documented submucosal ossification that can make complete endoscopic resection uncertain even when grossly en bloc, and (iii) the constrained anatomy of the appendiceal orifice. These factors collectively favored definitive surgical management.

Cross-sectional imaging (computed tomography or magnetic resonance imaging) is not routinely required when HO is identified in an appendiceal-orifice polyp but may be considered when symptoms are present or when complete endoscopic removal is doubtful because of size, firmness, or prior incomplete resections.

Reported presentations of gastrointestinal HO are varied and include incidental endoscopic findings (most common), lower gastrointestinal bleeding, intestinal obstruction, perforation, and even clinical mimicry of acute appendicitis.^2–7^ Recognition of firmness or a gritty sensation during snare transection should alert the endoscopist to the possibility of HO and prompt efforts for complete en bloc removal and thorough histologic examination.

Clinical implications are direct. First, tactile clues such as firmness or a gritty transection surface should raise suspicion for HO and for a higher risk of incomplete resection. Second, ossified stroma can obscure or coexist with additional pathology, including small NETs, which reinforces the need for complete specimen retrieval and careful pathology review. Third, management should prioritize complete en bloc excision when safely feasible. For diminutive lesions that are amenable to endoscopic therapy, underwater cold-snare polypectomy is effective and concordant with recommendations for SSLs up to 10 mm when margins can be assessed reliably.^8^ When complete endoscopic removal is uncertain, surgical resection is appropriate.

The long-term behavior of SSLs with HO is unknown. Until more evidence accrues, applying standard SSL surveillance intervals after complete excision is reasonable. Our case supports a practical approach in the appendiceal orifice: treat firmness as a marker of higher technical risk and ensure thorough histologic evaluation to avoid missed synchronous neoplasia.

## DISCLOSURES

Author contributions: PJ Wurtz: conception, literature review, manuscript drafting, and critical revision. D. Walton: literature review, histopathology analysis, and figure preparation. C. Gist & S. Murphy: literature review, surgical management, and critical revision. C. Kay: literature review, manuscript drafting, endoscopic management, and supervision. All authors reviewed and approved the final manuscript and agree to be accountable for all aspects of the work. PJ Wurtz is the article guarantor.

Financial disclosure: The authors declare no conflicts of interest relevant to this work.

Previous presentation: Presented as a poster at Digestive Disease Week (DDW) 2025, May 3–6, 2025, San Diego, CA.

Informed consent was obtained for this case report.

Disclaimer: The views expressed are those of the author(s) and do not reflect the official policy or position of Brooke Army Medical Center, the Department of Defense, or any agencies under the U.S. Government.

## ABBREVIATIONS:

BMP: bone morphogenetic protein
HO: heterotopic ossification
INSM1: insulinoma-associated protein 1
NET: neuroendocrine tumor
SSL: sessile serrated lesion
WHO: World Health Organization
